# PPARγ Gene Polymorphisms, Metabolic Disorders, and Coronary Artery Disease

**DOI:** 10.3389/fcvm.2022.808929

**Published:** 2022-03-23

**Authors:** Yongyan Song, Shujin Li, Chuan He

**Affiliations:** ^1^Central Laboratory, Clinical Medical College and Affiliated Hospital of Chengdu University, Chengdu, China; ^2^Department of Cardiology, Clinical Medical College and Affiliated Hospital of Chengdu University, Chengdu, China

**Keywords:** peroxisome proliferator-activated receptor gamma, PPARγ, *PPARG*, polymorphism, coronary artery disease

## Abstract

Being activated by endogenous and exogenous ligands, nuclear receptor peroxisome proliferator-activated receptor gamma (PPARγ) enhances insulin sensitivity, promotes adipocyte differentiation, stimulates adipogenesis, and has the properties of anti-atherosclerosis, anti-inflammation, and anti-oxidation. The Human PPARγ gene (*PPARG*) contains thousands of polymorphic loci, among them two polymorphisms (rs10865710 and rs7649970) in the promoter region and two polymorphisms (rs1801282 and rs3856806) in the exonic region were widely reported to be significantly associated with coronary artery disease (CAD). Mechanistically, *PPARG* polymorphisms lead to abnormal expression of *PPARG* gene and/or dysfunction of PPARγ protein, causing metabolic disorders such as hypercholesterolemia and hypertriglyceridemia, and thereby increasing susceptibility to CAD.

## Introduction

Coronary artery disease (CAD) is the most common type of cardiovascular disease globally, and is often caused by stenosis of coronary arteries due to atherosclerosis (1, 2). Under the action of various cardiovascular risk factors, atherosclerotic plaques gradually form, enlarge and ultimately block blood-vessel cavity, resulting in myocardial ischemia, hypoxia, and necrosis (3). According to the summary of the 2018 Report on Cardiovascular Diseases in China, cardiovascular disease is currently ranked as the first cause of death in China, and the morbidity and mortality are still on the rise (4). As a complex disease with multiple risk factors and being closely related to glucose and lipid metabolism, genetic variations in metabolism-related genes play an essential role in the pathogenesis of CAD (5). In recent decades, more and more genetic susceptibility genes and polymorphic loci for cardiovascular disease were explored and identified (6). Peroxisome proliferator-activated receptors (PPARs) are ligand-inducible transcription factors, belonging to the nuclear receptor superfamily (7). PPARs have multiple and complex physiological functions, involving lipid and glucose metabolism, (8, 9) inflammatory response, (10, 11) oxidative stress, (11, 12) cell differentiation and apoptosis, (13, 14) and even cognitive function (15). PPARs have been implicated in the pathogenesis of a number of major human diseases, such as cardiovascular and cerebrovascular diseases, (16, 17) malignant tumors, (18, 19) diabetes mellitus, (20, 21) metabolic syndrome, (22) and neurodegenerative disorders (23). PPARs have three subtypes: PPARα, PPARβ/δ, and PPARγ. Among them, PPARγ has the most in-depth exploration. According to NCBI’s reference sequence (RefSeq) database^1^, eight PPARγ isoforms have been identified in humans. Being activated by endogenous and exogenous ligands, PPARγ works in concert with retinoid X receptor (RXR) and is able to increase the insulin sensitivity, (24) promote adipocyte differentiation, (25) and stimulate adipogenesis, (26) and has the properties of anti-atherosclerosis, (27, 28) anti-inflammation, (27, 28) and anti-oxidation (29). This review focuses on recent progress in the association studies between polymorphisms in PPARγ gene (*PPARG*) and CAD, as well as the underlying mechanisms.

## PPARγ Gene and Its Polymorphisms

**FIGURE 1:**
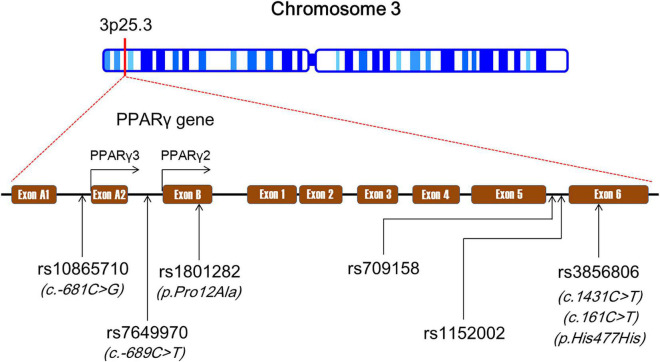
The genomic landscape of single-nucleotide polymorphisms (SNPs) in peroxisome proliferator-activated receptor gamma gene (*PPARG*). SNPs, single-nucleotide polymorphisms; *PPARG*, peroxisome proliferator-activated receptor gamma gene.

Human *PPARG* is located on the chromosome 3p25.3 and composed of nine exons: exon A1, exon A2, exon B, and exons 1–6 (Figure 1). There is alternative splicing in the maturation process of *PPARG* mRNA (30). According to the NCBI’s RefSeq database, sixteen PPARG mRNA splice variants have been identified so far in humans due to differential promoter usage and alternative splicing. *PPARG* is highly polymorphic (31). A large number of *PPARG* genetic variants have been recorded in the NCBI’s dbSNP database,^2^ and most of which are distributed in the intronic region. These loci can be divided into promoter region polymorphisms (e.g., rs10865710 and rs7649970), (32, 33) exonic region polymorphisms (e.g., rs1801282 and rs3856806), (32, 33) and intronic region polymorphisms (e.g., rs1152002 and rs709158) (33, 34) according to their distribution in the gene.

## Progress in the Association Studies Between PPARγ Gene Polymorphisms and Coronary Artery Disease

A large body of evidence indicated that some polymorphisms in *PPARG* are associated with CAD (34–63). Among them, rs1801282 (35, 41–51) and rs3856806 (52–63) in the exonic region were extensively reported to be significantly associated with CAD.

### Polymorphisms in the Promoter Region of PPARγ Gene and Coronary Artery Disease

Two promoter polymorphisms rs10865710 (35–37) and rs7649970 (38, 39) have been indicated to be correlated with CAD. Some other promoter variants such as c.93640T > C, c.93673T > C, and c.93695C > T have been investigated as well, and among them c.93695C > T was detected to be significantly associated with CAD (40).

#### The rs10865710 Polymorphism and Coronary Artery Disease

The rs10865710 polymorphism (also known as c.-681C > G) is located in the upstream promoter region of PPARγ3 gene and formed by a transversion from cytosine (C) to guanine (G) (35–37) (Figure 1). According to the NCBI’s dbSNP and VannoPortal^3^ databases, G is the minor allele of the rs10865710 polymorphism with frequencies ranging from 0.23 to 0.33 among Caucasian populations, 0.25 to 0.36 among Asian populations, and 0.21 to 0.24 among African populations. The results of two case–control studies (35, 36) in Chinese populations suggested that G allele of the rs10865710 polymorphism was associated with an increased risk of CAD. Zhang et al. (35) found that G allele carriers of the rs10865710 polymorphism had a higher risk of CAD than non-carriers in a Chinese population (odds ratio [*OR*], 1.47; 95% *CI*, 1.15–1.92; *p* < 0.001). Ding et al. (36) confirmed this finding in another Chinese population, and demonstrated that G allele of the rs10865710 polymorphism was associated with a higher risk of CAD (*OR*, 1.31; 95% *CI*, 1.16–1.95; *p* < 0.01). Our research team recently conducted a case–control study to assess the association between the rs10865710 polymorphism and CAD severity among Chinese patients, and observed that G allele carriers had higher Gensini scores (an indicator of CAD severity) (*p* < 0.05) and more diseased coronary branches than patients with CC genotype (*p* < 0.05) (37). However, several case–control studies carried out in Caucasians did not detect any significant association between the rs10865710 polymorphism and CAD risk (40, 60, 64). Hence, the impact of the rs10865710 polymorphism on susceptibility to CAD remains undetermined, and it may be modulated by ethnicity, living region, and/or eating habits. More studies are needed to elucidate the relationship of the rs10865710 polymorphism to CAD.

#### The rs7649970 Polymorphism and Coronary Artery Disease

The rs7649970 polymorphism (also known as c.-689C/T) is located in the upstream promoter region of PPARγ2 gene and formed by a transition from C to thymine (T) (38, 39) (Figure 1). According to the NCBI’s dbSNP and VannoPortal databases, T is the minor allele of the rs7649970 polymorphism with frequencies ranging from 0.11 to 0.17 among Caucasian populations, 0.03 to 0.06 among Asian populations, and 0.17 to 0.19 among African populations. A couple of case–control studies demonstrated that T allele of the rs7649970 polymorphism is associated with a higher risk of CAD (38, 39). Li et al. (38) examined the relationship between the rs7649970 polymorphism and CAD in a non-diabetic Chinese Han population, and the results showed that T allele is an independent risk factor for CAD after adjustment for conventional risk factors such as smoking, hypertension, and dyslipidemia (*OR*, 1.67; 95% *CI*, 1.03–2.71; *p* = 0.04). In another Chinese population, T allele of the rs7649970 polymorphism was found to be an independent risk factor for myocardial infarction (MI) after adjustment for traditional risk factors (*OR*, 2.13; 95% *CI*, 1.21–3.74; *p* < 0.01) (39). Dallongeville et al. (60) observed that TT genotype of the rs7649970 polymorphism was correlated with a marginally insignificantly higher risk of CAD in a large group of middle-aged men recruited from Lille, Strasbourg, and Toulouse in France and Belfast in Northern Ireland (*OR*, 3.34; 95% *CI*, 0.98–11.45; *p* = 0.05).

#### Other Polymorphisms in the Promoter Region and Coronary Artery Disease

Relationships of several rare variants in *PPARG* such as c.25924C > T and c.26233T > A in PPARγ3 promoter, and c.93640T > C, c.93673T > C, and c.93695C > T in PPARγ4 promoter to CAD were explored in an Italian population, and the explorers found that the c.93695C > T polymorphism was significantly correlated with acute coronary syndrome (ACS); T allele conferred a protective effect against ACS at both univariate (*OR*, 0.45; 95% *CI*, 0.29–0.69; *p* < 0.001) and multivariate (*OR*, 0.44; 95% *CI*, 0.25–0.76; *p* < 0.01) analyses (40).

### Polymorphisms in the Exonic Region of PPARγ Gene and Coronary Artery Disease

The association studies between polymorphisms in the exonic region of *PPARG* and CAD were heavily focused on the rs1801282 (35, 41–51, 60, 65–78) and rs3856806 (52–64, 79–86) polymorphisms. Other *PPARG* exonic polymorphisms in the association studies with CAD were scarcely investigated and rarely reported in the literature.

#### The rs1801282 Polymorphism and Coronary Artery Disease

The rs1801282 polymorphism (also known as p.Pro12Ala) is located in exon B of *PPARG* and is a missense variant in PPARγ2 resulting in a proline-to-alanine substitution (41–51) (Figure 1). This polymorphism is formed by a single-nucleotide change from C to G. G is the minor allele of the rs1801282 polymorphism with frequencies ranging from 0.11 to 0.17 among Caucasian populations, 0.02 to 0.06 among Asian populations, and 0.01 to 0.02 among African populations according to the NCBI’s dbSNP and VannoPortal databases. Researchers from various laboratories around the world have suggested that G allele of the rs1801282 polymorphism was associated with a higher risk of CAD (35, 41–46). Zhang et al. (35) evaluated the association between the rs1801282 polymorphism and CAD risk in a hospital-based study in Beijing, China, and observed that G allele carriers had a higher risk of CAD than non-carriers (*OR*, 1.69; 95% *CI*, 1.27–2.09; *p* < 0.001). In a case–control study carried out in Inner Mongolia, China, the investigators concluded that G allele of the rs1801282 polymorphism was an independent risk factor for MI after adjustment for conventional risk factors (*OR*, 2.68; 95% *CI*, 1.04–6.95; *p* = 0.04) (42). Hasan et al. (43) demonstrated that G allele carriers of the rs1801282 polymorphism were three times more likely to have CAD than non-carriers (*OR*, 3.0; 95% *CI*, 1.5–6.0; *p* = 0.001) among Egyptian patients with type 2 diabetes mellitus (T2DM). Maciejewska-Skrendo et al. (44) found that patients with unstable angina had a higher frequency of G allele of the rs1801282 polymorphism than healthy controls among European Caucasians (17.28 *vs*. 9.26%; *p* < 0.001). The association between the rs1801282 polymorphism and CAD appeared to be gender-dependent. Vogel et al. (45) demonstrated that GG genotype of the rs1801282 polymorphism was associated with a higher risk of ACS among Danish men (hazard ratio [*HR*], 2.12; 95% *CI*, 1.00–4.48; *p* = 0.05), but not among women. Similarly, Schneider et al. (46) observed that G allele of the rs1801282 polymorphism was significantly associated with CAD severity among German male patients (β, 0.32; *p* = 0.001). In a prospective cohort study involving middle-aged French men, the subjects with GG genotype of the rs1801282 polymorphism had a marginally insignificantly higher risk of CAD than those with CC genotype (*OR*, 3.32; 95% *CI*, 0.97–11.39; *p* = 0.06) (60).

Somehow, several case–control and prospective cohort studies have come to an opposite conclusion, demonstrating that G allele of the rs1801282 polymorphism was significantly associated with a reduced risk of CAD (47–51). Ho et al. (47) conducted a prospective case–control study among Hong Kong Chinese patients with T2DM and found that patients with CC genotype of the rs1801282 polymorphism had a higher risk of CAD than G allele carriers (*HR*, 4.38; 95% *CI*, 1.03–18.57; *p* = 0.05). A few studies involving American and European Caucasians confirmed that G allele of the rs1801282 polymorphism was associated with a lower risk of CAD (48–50). In an African population, Youssef and teammates (51) demonstrated that ACS patients had a lower frequency of G allele than control subjects (12.3 *vs*. 19.3%; *p* < 0.01), and the ACS patients carrying one or two G alleles had lower Gensini scores (*p* < 0.001) and less number of diseased coronary arteries (*p* < 0.001) than those with CC genotype.

Many well-designed studies have failed to detect a significant association between the rs1801282 polymorphism and CAD (65–76). Three case–control studies, respectively, conducted in Zhejiang University, (65) Shanghai Jiao Tong University, (66) and Chinese Medical University in China (67) could not find any significant association between the rs1801282 polymorphism and CAD. Furthermore, no significant association was detected between the rs1801282 polymorphism and CAD in various populations such as British, (68) Germans, (69) Canadians, (70) Koreans, (71) Turks, (72, 73) Dutch, (74) Indians, (75) and Thais (76).

The inconsistencies and contradictions among the association studies between the rs1801282 polymorphism and CAD may be due to the interaction of *PPARG* polymorphisms with environmental factors on cardiovascular risk factors, i.e., different alleles have different impacts on the expression patterns of *PPARG* under different environmental conditions. Abaj et al. (87) examined the interaction of the rs1801282 polymorphism with diet indices such as Dietary Quality Index-International, dietary phytochemical index, and healthy eating index on cardiovascular risk factors in T2DM patients, and found that these diet patterns did have a significant impact on cardiovascular risk factors in patients with different rs1801282 genotypes. In addition, small sample size, racial differences, and population heterogeneity may also be responsible for the inconsistencies and contradictions among studies. A meta-analysis combines data of the same type of studies to reduce the impact of confounding factors such as sample size and ethnicity on research results; so conclusions from meta-analyses are relatively more reliable. Wu and teammates (77) performed a meta-analysis with 22 studies and 23,375 subjects enrolled, and found that GG genotype of the rs1801282 polymorphism conferred a higher risk of CAD than CC genotype in the total population (*OR*, 1.30; 95% *CI*, 1.01–1.68; *p* = 0.04) and in Caucasians (*OR*, 1.44; 95% *CI*, 1.07–1.93; *p* = 0.02), but not in Asians. However, the results from several other meta-analyses did not support this finding and concluded that the rs1801282 polymorphism was not associated with CAD in overall and subgroup analyses (60, 61, 78). Therefore, it is difficult to reach a consistent conclusion referring to the relationship between the rs1801282 polymorphism and CAD based on the existing research data. Further studies are needed to clarify this issue.

#### The rs3856806 Polymorphism and Coronary Artery Disease

The rs3856806 polymorphism (also known as c.1431C > T, c.161C > T, or p.His477His) is located in exon 6 of *PPARG* and is a synonymous variant in PPARγ2 (52–64) (Figure 1). The c.1431C > T is named according to the position of this variant in PPARγ2 cDNA, as this variant is located at 1431 bp downstream of the start codon (ATG). This c.161C > T is defined based on the position of this variant in exon 6 of *PPARG* gene since it is located at 161 bp downstream of the first nucleotide of exon 6 of *PPARG*. The rs3856806 polymorphism is formed by a single-nucleotide substitution from C to T. T is the minor allele of the rs3856806 polymorphism with frequencies ranging from 0.10 to 0.22 among Caucasian populations, 0.18 to 0.28 among Asian populations, and 0.05 to 0.07 among African populations according to the NCBI’s dbSNP and VannoPortal databases. A number of case–control and cross-sectional studies have shown that T allele of the rs3856806 polymorphism was associated with a reduced risk of CAD and was a protective allele for CAD (52–59). In a hospital-based case–control study of Chinese patients with CAD and chest pain syndrome, Liu et al. (52) found that the T allele carriers of the rs3856806 polymorphism had a reduced CAD risk compared with CC homozygotes (*OR*, 0.55; 95% *CI*, 0.33–0.83; *p* = 0.01). Zhou and the other two teammates (53) made a similar finding in the Chinese Han population that the T allele carriers of the rs3856806 polymorphism had a 39% decreased risk of CAD relative to CC homozygotes (*OR*, 0.61; 95% *CI*, 0.49–0.76; *p* < 0.001). The protective effect of T allele on CAD was also reported by other explorers in Chinese populations (54–59). It seems that the correlation between the rs3856806 polymorphism and CAD is stronger in the presence of T2DM as Wan et al. (58) observed that the T allele was significantly correlated with a lower degree of coronary stenosis (<75%) among CAD patients combined with T2DM (*p* = 0.02), but not among patients free of T2DM (*p* = 0.70).

Like the rs1801282 polymorphism, there was also a contradiction in the relation between the rs3856806 polymorphism and CAD. Several studies suggested that the T allele of the rs3856806 polymorphism was associated with an increased risk of CAD (40, 60–63). Chao et al. (62) demonstrated that the TT genotype conferred a higher risk of MI as compared to CC genotype in a Taiwanese population (*OR*, 2.7; 95% *CI*, 1.1–6.5). A significant association between T allele of the rs3856806 polymorphism and higher risk of ACS was detected among Chinese mainland residents (*OR*, 1.63; 95% *CI*, 1.00–2.65; *p* = 0.05) (61) and Italians (*p* = 0.03) (40). In a French male population, TT homozygotes had a higher risk of CAD than CC homozygotes (*OR*, 5.93; 95% *CI*, 1.19–29.45; *p* = 0.03), (60) and in an Iranian population, T allele carriers had a higher risk of CAD than CC homozygotes (*OR*, 2.28; 95% *CI*, 1.20–4.35; *p* = 0.01) (63). Recent experimental results from our laboratory showed that the T allele of the rs3856806 polymorphism was correlated with an increased risk of T2DM complicated with CAD (*p* = 0.03) (37).

Some researchers failed to detect any significant association of the rs3856806 polymorphism with CAD (64, 65, 79–82). Results from three independent studies conducted in China consistently indicated that the rs3856806 polymorphism was not correlated with CAD (65, 79, 80). Neither Yilmaz-Aydogan (81) nor Yongsakulchai (82) did in a Turkish population and in a Thai population, respectively. We did not find any significant association of the rs3856806 polymorphism with CAD or CAD severity as well (37).

A couple of meta-analyses have been carried out in order to clarify the relationship between the rs3856806 polymorphism and CAD, but still no consistent results were obtained to date. Qian et al. (61) did a meta-analysis with 9 studies and a total of 3,878 subjects enrolled, and the result suggested that T allele carriers of the rs3856806 polymorphism had a lower CAD risk than CC homozygotes (*OR*, 0.69; 95% *CI*, 0.59–0.82; *p* < 0.001). Gonzlez-Castro et al. (83) expanded the sample size to 21 studies and 15,980 subjects, and arrived at a similar conclusion (*OR*, 0.33; 95% *CI*, 0.20–0.52). However, Ding et al. (78) demonstrated that T allele carriers of the rs3856806 polymorphism had a higher risk of CAD than non-carriers by meta-analysis (*OR*, 1.18; 95% *CI*, 1.02–1.34; *p* < 0.01). In addition, the results from several other meta-analyses indicated that the rs3856806 polymorphism was not correlated with CAD at all (84–86). Hence, the relationship between the rs3856806 polymorphism and CAD is not possible to be determined based on the existing research data, and it needs to be further explored.

### Polymorphisms in the Intronic Region of PPARγ Gene and Coronary Artery Disease

A few studies have been carried out to explore the associations between *PPARG* intronic polymorphisms and CAD. The rs1152002 polymorphism is located in intron 5 of *PPARG* and formed by a transition from G to adenine (A). Tian et al. (34) reported that A allele of the rs1152002 polymorphism was associated with a higher risk of CAD in a Chinese population (*OR*, 2.92; 95% *CI*, 1.44–5.94; *p* < 0.01). The rs709158 polymorphism is located in intron 5 of *PPARG* and formed by a transition from A to G. Gallicchio et al. (88) prospectively examined the association of the rs709158 polymorphism with cardiovascular morbidity and mortality in a community-based cohort study, and demonstrated that there was no statistically significant association between them.

## Mechanisms Underlying the Associations Between PPARγ Gene Polymorphisms and Coronary Artery Disease

**FIGURE 2:**
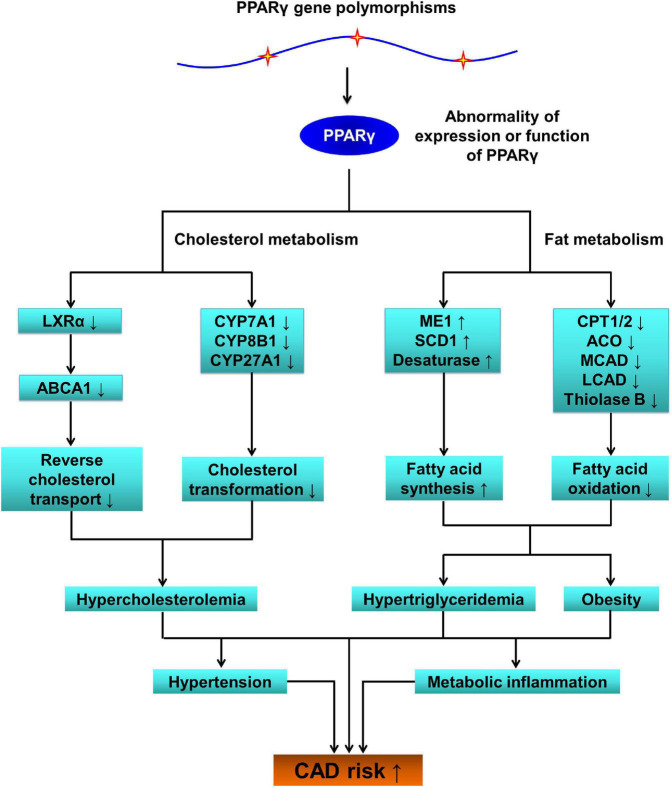
The pathophysiological role of *PPARG* SNPs in coronary artery disease (CAD). *PPARG*, peroxisome proliferator-activated receptor gamma gene; PPARγ, peroxisome proliferator-activated receptor gamma; LXRα, liver X receptor alpha; ABCA1, ATP-binding cassette transporter A1; CYP7A1, cytochrome P450 family 7 subfamily A member 1; CYP8B1, cytochrome P450 family 8 subfamily B member 1; CYP27A1, cytochrome P450 family 27 subfamily A member 1; ME1, malic enzyme 1; SCD1, stearoyl-coenzyme A desaturase 1; CPT1/2, carnitine palmitoyltransferase 1/2; ACO, acyl-coenzyme A oxidase; MCAD, medium-chain acyl-coenzyme A dehydrogenase; LCAD, long-chain acyl-coenzyme A dehydrogenase; SNPs, single-nucleotide polymorphisms; CAD, coronary artery disease.

In terms of mechanisms of action by which *PPARG* polymorphisms influence on the susceptibility to CAD, the first thing that comes to mind is that polymorphisms in *PPARG* lead to abnormal expression of this gene and/or dysfunction of PPARγ protein, resulting in aberrant expressions of PPARγ-targeted genes, metabolic disorders, and arteriosclerotic cardiovascular disease (Figure 2).

### PPARγ Gene Polymorphisms and Gene Expression Efficiency

Being activated by endogenous and exogenous ligands, PPARγ mainly up-regulates gene expressions of enzymes and transporters that play key roles in lipid and glucose metabolic pathways such as reverse cholesterol transport, (89, 90) cholesterol transformation, (89, 90) lipogenesis, (91, 92) fatty acid oxidation, (93, 94) and gluconeogenesis (95). By using luciferase reporter and electrophoretic mobility shift assays, Lu et al. (96) observed that G allele of the rs10865710 polymorphism significantly inhibited the DNA-binding activity of transcription factor cAMP-response element-binding protein 2 (CREB2) to PPARγ3 promoter. The rs948820149 polymorphism (c.-807A > C) is located in PPARγ2 promoter and C allele was found to significantly down-regulate PPARγ2 expression by affecting the DNA-binding activity of transcription factor glucocorticoid receptor β (GRβ) to PPARγ2 promoter (97). Another two *PPARG* promoter polymorphisms c.-1633C > T and c.-1572G > A were verified to modulate the expression efficiency of *PPARG* in Erhualian pigs as well (98). Pihlajamäki et al. (99) compared PPARγ2 mRNA expression as well as its two target genes (lipid phosphate phosphohydrolase 1 [LPIN1] and sterol-regulatory-element-binding protein 1c [SREBP-1c]) between *PPARG* rs1801282 genotypes in human adipose tissues, and observed that Ala12Ala genotype was associated with a significantly higher mRNA expression compared to Pro12Pro genotype. By using a computational analysis of SNPs in *PPARG*, researchers found that mutations in *PPARG* impaired functions of PPARγ, leading to serious complications such as obesity, diabetes, and cancer in humans (31).

It is easy to understand that *PPARG* polymorphisms in the promoter region, as well as missense polymorphisms in the exonic region, may cause metabolic disorders such as hypercholesterolemia, hypertriglyceridemia, and hyperglycemia, which subsequently increase the risk of CAD. However, it is difficult to explain how intronic and synonymous polymorphisms are responsible for susceptibility to CAD. So far, there is no direct evidence that *PPARG* intronic and synonymous polymorphisms modulate *PPARG* gene expression efficiency. Little is known about the molecular mechanisms underlying the regulatory function of intronic and synonymous polymorphisms in *PPARG* on its gene expression, but several possible explanations can be put forward for intronic polymorphisms. Firstly, there are functional elements in intronic regions to regulate gene expression, such as intronic enhancer/repressor (100, 101). Secondly, intronic polymorphisms may affect the pre-mRNA splicing process (102). Thirdly, some non-coding RNAs with a wide range of regulatory effects are encoded by introns (103, 104). Regarding synonymous polymorphisms, they may alter the secondary structure of pre-mRNA, and thereby influencing mRNA splicing efficiency and protein translation (105–107).

### PPARγ Gene Polymorphisms and Plasma Lipid Levels

Dyslipidemia is a major risk factor for CAD, accounting for 50% of the population attributable risk (108). Increases in the levels of triglycerides, total cholesterol and low-density lipoprotein cholesterol (LDL-C), and/or decreases in HDL-C levels confer a high risk of CAD. There is accumulating evidence indicating that *PPARG* exonic polymorphisms rs1801282 (41–43, 64–66, 109–112) and rs3856806 (37, 53, 62, 64, 79, 112) are significantly associated with abnormal levels of plasma lipids. The *PPARG* promoter polymorphisms rs10865710 and rs7649970 have been reported to be significantly correlated with plasma lipid levels as well, (37–39) although there were few studies conducted in the scientific community.

#### The rs1801282 Polymorphism and Plasma Lipid Levels

A number of observational studies suggested that G allele of the rs1801282 polymorphism was associated with increased levels of triglycerides, (110, 112) total cholesterol, (41–43, 64–66, 109) and LDL-C, (43, 64, 65, 109) and decreased levels of HDL-C, (42, 43) which is in line with the finding of several case–control studies that G allele carriers had a significantly higher risk of CAD than CC homozygotes (41–46). In a Chinese population, Wang et al. (42) found that G allele carriers of the rs1801282 polymorphism had significantly higher levels of total cholesterol and LDL-C than the subjects with CC genotype, and also that G allele carriers were at a higher risk of MI. Similarly, Hasan et al. (43) demonstrated that G allele carriers of the rs1801282 polymorphism had significantly higher levels of total cholesterol and LDL-C, and lower levels of HDL-C than CC homozygotes in an Egyptian diabetic population, and the researchers also observed that the risk of CAD was three times higher among G allele carriers than among non-carriers.

Just as there were contradictions in the associations between the rs1801282 polymorphism and CAD, some notable inconsistencies were present in the relations between the rs1801282 polymorphism and plasma lipid levels. In a Chinese longevity population (age >90 years), the levels of total cholesterol, LDL-C, and HDL-C were comparable between the rs1801282 genotypes (CG + GG *vs*. CC), but G allele carriers had significantly lower levels of triglycerides than the subjects with CC genotype (*p* < 0.001) (110). Koohdani et al. (111) also reported lower levels of triglycerides in G allele carriers of the rs1801282 polymorphism than in CC homozygotes among Iranian T2DM patients.

#### The rs3856806 Polymorphism and Plasma Lipid Levels

Several studies demonstrated that the T allele of the rs3856806 polymorphism was correlated with decreased levels of triglycerides, (64) total cholesterol, (112) and LDL-C, (112) as well as elevated levels of HDL-C (53). This may explain the phenomenon that the T allele was associated with a reduced risk of CAD in several case–control studies (52–59). Zhou et al. (53) observed that the T allele carriers of the rs3856806 polymorphism had lower levels of triglycerides, total cholesterol, and LDL-C, higher levels of HDL-C, and a 40% lower risk of CAD than non-carriers in the Chinese Han population. In the Russian population, the investigators reported that serum levels of triglycerides in T allele carriers of the rs3856806 polymorphism were significantly lower than in the subjects with CC genotype, and simultaneously the frequency of T allele tended to decrease in CAD patients compared to control subjects (64).

A few studies have produced conflicting results that the T allele of the rs3856806 polymorphism was correlated with higher levels of atherogenic lipids (37, 62, 79). Chao et al. (62) reported that the T allele carriers had significantly higher levels of oxidized low-density lipoprotein (an atherogenic lipoprotein) than the subjects with CC genotype in a Taiwanese population, and accordantly TT homozygotes were found to have a significantly higher risk of MI than C carriers. In a group of Chinese patients with CAD, our research team found that the T allele carriers of the rs3856806 polymorphism had significantly higher levels of total cholesterol, LDL-C and apolipoprotein B than non-carriers (37). In addition, triglycerides, very-low-density lipoprotein cholesterol (VLDL-C) and lipoprotein (a) were found to be higher in T allele carriers of the rs3856806 polymorphism than in non-carriers in a hospital-based study (79).

#### The rs7649970 and rs10865710 Polymorphisms and Plasma Lipid Levels

A couple of studies (38, 39) showed that the T allele of the rs7649970 polymorphism was correlated with increased levels of triglycerides and total cholesterol, and T allele frequency was also higher in CAD patients than in control subjects. We found that G allele carriers of the rs10865710 polymorphism had significantly higher levels of atherogenic lipids such as total cholesterol, lipoprotein (a) and apolipoprotein B, higher Gensini scores, and more diseased coronary branches (37).

### PPARγ Gene Polymorphisms and Blood Pressure

Hypertension is a recognized risk factor for CAD. Ettehad et al. (113) reported that the risk of CAD decreased by 17% for every 10 mmHg reduction in systolic blood pressure. One population-based study (110) and three meta-analyses (114–116) collectively pointed out that G allele of the rs1801282 polymorphism was associated with significantly reduced blood pressure. It is consistent with the finding that G allele of the rs1801282 polymorphism was correlated with a reduced risk of CAD in case–control studies (47–51). Lu et al. (110) reported that G allele of the rs1801282 polymorphism appeared to have a protective effect against hypertension. This finding was validated by Regieli et al. in the Dutch population (50) and by three meta-analyses (114–116). However, some other studies had a completely different finding (41, 43). Li et al. (41) suggested that G allele carriers of the rs1801282 polymorphism had significantly higher systolic blood pressure than non-carriers in patients with MI, and accordingly, the frequency of G allele was significantly higher in MI patients than in healthy subjects. Hasan et al. (43) demonstrated that G allele of the rs1801282 polymorphism was significantly correlated with increased systolic and diastolic blood pressure compared to C allele among Egyptian T2DM patients, and as well G allele carriers had a significantly higher risk of CAD. Regarding the rs3856806 polymorphism, our research team found that T allele carriers had significantly higher systolic and diastolic blood pressure than CC homozygotes among CAD patients (37).

### PPARγ Gene Polymorphisms and Obesity Indexes

Body mass index (BMI), waist-to-hip ratio (WHR), and waist circumference (WC) are common indicators of obesity and are closely related to CAD (117–119). Several studies have shown that G allele of the rs1801282 polymorphism was associated with higher BMI, (43, 66, 111, 120) WC, (43, 111) and WHR, (66) higher prevalence of central obesity and higher percentage of body fat (121). This can explain from one aspect why G allele of the rs1801282 polymorphism was correlated with increased risk of CAD in several case–control studies (41–46). The association between the rs1801282 polymorphism and BMI was validated by two meta-analyses which concluded that G allele carriers had significantly higher BMI and higher prevalence of obesity than the subjects with CC genotype (122, 123). However, some studies (124–126) have come to conflicting conclusions. da Silva et al. (124) demonstrated that CC homozygotes had significantly higher BMI and WHR compared to CG heterozygotes among Brazilian adult men. A similar finding was observed in teenagers from Northern Mexico, and the researchers noted that G allele carriers of the rs1801282 polymorphism exhibited significantly lower overweight/obesity phenotype (BMI *Z*-score) frequency than CC homozygotes (125). Zafar et al. (126) found that BMI and WC were significantly lower in GG homozygotes compared to CC homozygotes among patients with metabolic syndrome. The rs3856806 polymorphism was also reported to be significantly correlated with BMI in Chinese populations, and T allele carriers had significantly higher BMI than the subjects with CC genotype (37, 53).

### PPARγ Gene Polymorphisms and Other Cardiovascular Risk Factors

Atherosclerosis is actually an ongoing chronic inflammatory disorder, not just a simple lipid deposition on the intima and media walls of blood vessels (127, 128). A fundamental role of low-grade inflammation has been established in mediating all stages of atherosclerosis from the initiation to the formation of atherosclerotic plaques and ultimately to thrombosis in the blood vessels (129). Liu et al. (130) observed that T allele carriers of the rs3856806 polymorphism had significantly lower levels of C-reactive protein (CRP) than the subjects with CC genotype in hemodialysis patients. An interaction between the rs1801282 polymorphism and diet indices on cardiovascular risk factors was evaluated among patients with T2DM, and the highest IL-18 level was observed in G allele carriers with the highest adherence to Diet Quality Index (DQI) (76). Adiponectin has the properties of enhancing insulin sensitivity, (131) inhibiting inflammation, (132) and attenuating atherosclerosis (133). Campos-Perez et al. (134) reported that G allele carriers of the rs1801282 polymorphism had significantly higher levels of serum adiponectin than CC homozygotes in a general population, whereas Baldani et al. (135) could not find any significant correlation between the rs1801282 polymorphism and serum adiponectin levels among women with polycystic ovary syndrome.

## Conclusion and Prospect

With the increase of morbidity and mortality in patients with CAD in recent decades, researchers from all over the world have carried out a large number of observational and experimental studies on the associations between *PPARG* polymorphisms and CAD. Two polymorphic loci (rs1801282 and rs3856806) in the exonic region of *PPARG* were extensively explored in various populations and were reported to be significantly associated with CAD, but the risk alleles of these polymorphic loci are still elusive. In the future, multi-center, multi-ethnic, and large-sample case–control and cohort studies are needed to identify *PPARG* risk alleles for CAD. In addition, great efforts are required to assess the interactions between *PPARG* polymorphisms and environmental factors on the expression patterns of *PPARG*, the function of PPARγ, and the susceptibility to CAD.

## Author Contributions

All authors retrieved the literature and wrote and organized the manuscript.

## Conflict of Interest

The authors declare that the research was conducted in the absence of any commercial or financial relationships that could be construed as a potential conflict of interest.

## Publisher’s Note

All claims expressed in this article are solely those of the authors and do not necessarily represent those of their affiliated organizations, or those of the publisher, the editors and the reviewers. Any product that may be evaluated in this article, or claim that may be made by its manufacturer, is not guaranteed or endorsed by the publisher.

## Footnotes

1
http://www.ncbi.nlm.nih.gov/RefSeq/

2
http://www.ncbi.nlm.nih.gov/SNP/

3
http://www.mulinlab.org/vportal
